# Investigating the Interplay Between Participation in a STEM-Focused Student Success Program and Workforce Participation on STEM Undergraduate Degree Completion

**DOI:** 10.3389/fsoc.2022.818032

**Published:** 2022-04-28

**Authors:** Dina Ghazzawi, Donna Lynn Pattison, Catherine Lynn Horn

**Affiliations:** ^1^Department of Higher Education Leadership and Policy Studies, University of Houston, Houston, TX, United States; ^2^Department of Biology and Biochemistry, University of Houston, Houston, TX, United States; ^3^Department of Educational Leadership and Policy Studies, University of Houston, Houston, TX, United States

**Keywords:** STEM education, persistence, under-represented minorities, intervention, financial aid

## Abstract

This study longitudinally tracks students participating in a STEM-focused intervention program to investigate workforce participation patterns and their association with degree completion in a STEM field. Using longitudinal data from the University of Houston's Education Research Center, this study examines the extent to which students participating in a STEM intervention program require additional work to fund tuition and other life expenses. Findings demonstrated a negative effect of workforce participation on college completion and showed that minority students were more likely to participate in the workforce while also receiving financial support from the STEM program compared to peers from other racial backgrounds. Results inform institutional and financial aid policies, as well as admission criteria as it relates to broadening access of under-represented students in STEM.

## Introduction

The topic of broadening access to STEM fields has prompted several national efforts to address the need to diversify and expand STEM participation at the undergraduate level (Smyth and McArdle, 2004; Baber, 2015). Disparities in STEM degree attainment across race and socio-economic status present a prevalent and significant concern for the nation's ability to maintain and promote a competitive, diverse STEM workforce (Chapa and De La Rosa, 2006; Carnevale et al., 2011; Xie et al., 2015). These concerns are especially relevant in the current higher education landscape, where rising tuition costs and funding limitations stifle the role of post-secondary education in improving social and economic upward mobility (Fenske et al., 2000).

The potential value of receiving an undergraduate degree, particularly in a STEM major is significant and associated with increased future earnings (Thomas, 2000; Xie and Killewald, 2012). However, limitations to the amount of funding students receive for their education, coupled with the recent stagnation in earnings, affects the ability of today's racially and economically diverse student population to reap the benefits of attaining a degree in a STEM field (Mitchell et al., 2019). Several additional reasons, along with the financial struggles associated with STEM degree attainment, contribute to the lower rates of degree attainment and enrollment of low-income minority students. These include, but are not limited to, the continuous pattern of rising tuition costs above the cost of living, decreases in state funding for higher education, and the increasing institutional shifts from need-based to merit-based aid (Bowen et al., 2009; Ma and Matea, 2021). Furthermore, recent data trends indicate that even with the support of financial and institutional aid, students from low-income backgrounds face an increased likelihood of incurring debt to afford college compared to their peers (Ma and Matea, 2021). Similarly, studies highlight the significance of financial aid in assisting students to devote their time fully to their studies, as minority students often have to work extra hours to finance their education, causing their study time to suffer (Gardner and Broadus, 1990; Curs and Harper, 2012).

The continuous pattern of attrition and drop-out rates among under-represented students has spurred several national intervention efforts aimed toward improving retention and persistence rates among this subpopulation of students (Riegle-Crumb and King, 2010; National Center for Science and Engineering Statistics, 2018). STEM intervention programs, in recent years, have become an important conduit through which students from under-represented backgrounds can access and complete under-graduate degrees in STEM (Walker et al., 2010; Estrada, 2014; Carpi et al., 2017). Primarily through providing academic assistance and financial aid, such programs assist in bridging persistent gaps in STEM degree attainment, wage gaps across race, gender and economic status (Carnevale et al., 2011). Despite supporting evidence of the success of STEM intervention programs at increasing academic performance, collegiate success and retention of under-represented minorities in STEM fields (e.g., Lee and Harmon, 2013; Jackson and Winfield, 2014; Ghee et al., 2016; Ghazzawi et al., 2021a,b), less is known about the sufficiency of the financial aid offered to students through these programs, as well as its association with persistence and graduation rates of URM students. Access to adequate financial aid is among the top five factors associated with minority persistence in STEM fields of study, yet recent studies demonstrate that institutional barriers to financial aid, as well as increasing tuition costs, has caused insufficiencies in the financial aid awarded to low-income students, causing them to struggle (Mitchell et al., 2019). In addition, a substantial body of research highlights the various barriers under-represented students face to enroll in, access and succeed in their higher education journey. These include inadequate academic preparation, financial struggles, recruitment policies and unwelcoming campus climates (May and Chubin, 2003; Crisp et al., 2009; Espinosa, 2011; Dika and D'Amico, 2016; Lisberg and Woods, 2018).

### The Houston-Louis Stokes Alliance for Minority Participation

As part of the significant national intervention efforts to reduce racial disparities in STEM degree achievement, the Houston-Louis Stokes Alliance for Minority Participation was established in 1998 as an effort to improve graduation and persistence rates among under-represented minority students in STEM fields. The alliance includes five higher education institutions that rely on empirical research on collaborative learning techniques, peer led team learning, and faculty mentorship to guide program components most beneficial to minority student success in STEM (Treisman, 1992; Ghazzawi et al., 2021b). Through offering financial aid and academic support, the program has successfully graduated 2,044 students with bachelor degrees since the first graduating cohort of 2004, 92% of which were in STEM fields of study, to a majority-minority student body (Ghazzawi et al., 2021a).

In terms of student recruitment, the H-LSAMP program pays particular attention in targeting students from low-performing high-schools through a special review process, yet also recruits high-performing minority students (Ghazzawi et al., 2021b). Funding levels for H-LSAMP participants is primarily obtained from institutional funds and grant funding through the National Science Foundation. During the 2020–2021 academic year, 152 students were supported by NSF funds with an average award of $2,954 per student, and 469 students were supported by institutional funds averaging at $1,571 per student (Bonsangue and Drew, 2021). Taking into consideration the increase in cost of living and stagnant wage earnings, the sufficiency of these funds, as well as the extent to which recruitment initiatives target those most in need, requires further examination.

## Purpose of the Study

Given the importance of financial aid in the success of under-represented student success and the significance of STEM intervention programs in boosting the academic success of URM students, this paper seeks to investigate the extent to which financial aid offered through STEM intervention programs contributes to the successful graduation of URM students in STEM. Specifically, this study examines the degree to which students enrolled in a STEM intervention program join the workforce while enrolled in their undergraduate degree to develop a deeper understanding of the external, socio-economic factors that could affect students' decision to enroll, succeed and successfully matriculate into the STEM workforce or a graduate degree in STEM. In response to the many concerns to broaden and diversify access to STEM fields, this study aims to strengthen the body of research regarding the funding practices of STEM intervention programs, particularly as these programs affect students from under-represented backgrounds' ability to enter and persist in STEM fields (Rincón and George-Jackson, 2016). Specifically, this paper addresses the following research questions:

1. What are the socio-demographic, pre-college and academic characteristics of students enrolled in the Houston-Louis Stokes Alliance for Minority Participation program?
2. To what extent does participation in the workforce while enrolled in the H-LSAMP program associate with successful graduation in STEM?

Findings from this study will inform institutional and financial aid policy as it pertains to undergraduate success and persistence of URM students in STEM. On a broader level, this study aims to contribute to the understanding of the degree to which STEM intervention programs, and their funding priorities, support national efforts to diversify and expand access to STEM fields. Given that STEM intervention programs are primarily designed to improve access and success of this subpopulation of students, results from this study also aim to highlight the degree to which such programs truly support equity and diversity in higher education to traditionally under-served populations and offer legitimacy for increased financial support of these students.

### Literature Review

A substantial body of research highlights the multiple barriers faced by under-represented students throughout K-12 and into their matriculation to college (Chen and Weko, 2009; National Assessment of Educational Progress, 2011; Institute For Broadening Participation, 2014). These barriers are varied and multi-faceted, yet contribute to a persistent pattern of attrition and drop-out among this sub-population of students (May and Chubin, 2003; Xie and Killewald, 2012; Institute For Broadening Participation, 2014). Reasons pertaining to the high drop-out rates among URM students include, but are not limited to, a lack of adequate pre-college academic preparations, college admission criteria, and financial aid (May and Chubin, 2003; Aschbacher et al., 2010; Walker et al., 2010). URM students are less likely to have access to rigorous high school curriculum, specifically in the areas of math and science, which in turn negatively impacts their chances of enrolling in, and succeeding in a STEM field of study (Riegle-Crumb and King, 2010; Engberg and Wolniak, 2013). These patterns are particularly prevalent among economically disadvantaged students, who are considerably less likely to enter in and complete a STEM degree (Trent et al., 2006). Along those lines, the collective disadvantage of inadequate pre-college preparation at the school level restricts whether under-represented minority students will consider and enroll into post-secondary education as a whole (Rincón and Lane, 2017).

Barriers to the access, enrollment and collegiate success of URM students in STEM exemplify the role that systemic racism plays in reinforcing inequitable admission policies, beliefs, and distribution of resources among minority students (Edge, 2020; McGee, 2020; O'Hara, 2020). Efforts to reform and diversify STEM education, thus far, have perpetuated inherent racist structures rather than addressing mechanisms through which institutions can challenge the discriminatory foundation of STEM programs and policies (McGee, 2020). In response to national calls to broaden and diversify the STEM undergraduate population, STEM intervention programs were established, in part, to provide academic support and financial aid to students from disadvantaged, minority backgrounds (Walker et al., 2010; Estrada, 2014; Carpi et al., 2017). Many of these programs offer students a collaborative learning environment, including faculty support, mentorship, and peer group learning which assist in promoting sense of belonging and academic integration among students, particularly from historically under-served backgrounds (Chang et al., 2014). Despite evidence of the positive role of STEM intervention programs at increasing the persistence, academic integration, and graduation of minority students in STEM fields (e.g., Duncan and Dick, 2000; Lane, 2016; Ghazzawi et al., 2021b), less is known about the role that financial aid plays in assisting program participants from disadvantaged backgrounds successfully graduate in a STEM field.

Literature emphasizes the importance of financial aid in helping students dedicate their time fully to their studies, as minority students often have to work extra hours to afford their education, negatively impacting their study time and academic achievement (Gardner and Broadus, 1990; Curs and Harper, 2012). Discourse in financial aid and economic literature examines the impact of financial aid policies on student success measures, particularly with the recent increase in the proportion of students receiving merit-based aid, as opposed to need-based aid (Doyle, 2006; Curs and Harper, 2012). One of the major criticisms of these trends is that financial aid is more likely to benefit students from middle and upper-income classes to a greater extent than those with increased socio-economic need (Cabrera et al., 1993). Alon and Tienda (2007) expand on these criticisms by discussing the notion of meritocracy in higher education, whereby students' individual talent and academic ability, measured by test scores and pre-college academic performance, take precedence over ascribed measure such as the need for academic and financial support. As a consequence, the role of STEM intervention programs in expanding opportunities for those traditionally under-represented students contrasts with the basic premise of meritocracy in higher education. The reliance on standardized test scores and competitive admission policies, in essence, contributes to the increasing social and economic inequities inherent in today's society (Walker et al., 2010; Rincón and Lane, 2017). With this evidence in mind, this study contributes to the existing body of literature on financial aid policies and their impact on the collegiate success of URM students by investigating longitudinal patterns of workforce participation among students from varying degrees of financial need.

### Methodology

This study uses longitudinal data from the University of Houston's Education Research Center, a rich data repository that combines data from various state-level sources, including the Texas Education Agency (TEA), Texas Higher Education Coordinating Board (THECB), and the Texas Workforce Commission (TWC). Such expansive data sources lend this study the ability to track students' educational trajectory over time, from high school into post-secondary education and, subsequently, into the workforce. The sample included in this study were undergraduate participants of the H-LSAMP intervention program admitted between the years 1999–2015 (*n* = 1,767). Student-level characteristics, such as pre-college admission criteria, age, racial background, and SAT scores were examined, as well as the proportion of students participating in the workforce during their undergraduate degree program.

### Data Analysis

Data analysis for this study consisted of both descriptive and inferential statistics. First, cross-tabulations and chi-square tests were used to investigate the proportion of students participating in the workforce across racial background, admission standards, and degree completion. Such descriptive data offered insight into the significance of differences between students participating in the workforce during their time in college across various socio-demographic and pre-college characteristics associated with persistence and graduation in STEM (Curs and Harper, 2012; Rincón and George-Jackson, 2016). Subsequently, a logistic regression model was conducted to measure the effect of workforce participation on degree completion for H-LSAMP participants. Control variables, including race, gender, admission standards, and time to graduation, were used in the analysis to examine the interplay between socio-demographic and pre-college factors, in association with workforce participation, and their influence on degree completion among historically under-served students.

### Variables

#### Dependent Variable

The dependent variable of interest in this analysis was degree completion, a dichotomous variable representing whether students graduated in the specific time-frame of the study, or have not yet graduated.

#### Independent Variables

Supported by research studies that associate socio-demographic and pre-college variables with student success, particularly for students of color, independent variables included in this analysis consisted of racial/ethnic background, pre-college SAT scores, gender, age, as well as workforce participation and industry.

### Limitations

There are a few limitations to this study that warrant discussion. Firstly, data on the specific amount of aid awarded to students, as well as the socio-economic status of students, was not available to use in the current study. Although this data would add valuable information on the relationship between socio-economic status, and financial aid rewarded, on the likelihood of working while studying and degree completion rates, this study used HLSAMP participation, and the increased likelihood of HLSAMP scholars being from economically disadvantaged backgrounds, as a proxy for socio-economic status and financial need. One of the major objectives of STEM intervention programs, particularly the program we focus on in this study, is to provide academic support and financial assistance to historically under-represented students without the financial resources or pre-college academic preparation needed to pursue an undergraduate degree in STEM (Chang et al., 2014; Carpi et al., 2017; Lisberg and Woods, 2018). In addition, specific information concerning the level of unmet financial need was not available for the purposes of this study. Despite the limitations in data availability, this study uses empirical research and HLSAMP recruitment objectives to discern the financial need of students participating in the program.

In addition, while the results of the study offer program administrators of intervention programs a breadth of information on the association between workforce participation during the HLSAMP fellowship and degree completion rates, it is important to note that these results are limited to the HLSAMP program and the efforts of participating institutions in Texas. The racial/ethnic composition of students graduating from the HLSAMP program thus far closely resembles that of the population demographics in Houston for Black students, as 26.9% of HLSAMP graduates were Black, compared to 22.6% of the Houston population (Cheeseman Day and Martinez, 2021; Ghazzawi et al., 2021b). However, Hispanic graduates are significantly less compared to population demographics (23.4 vs. 45%). Therefore, given the scope of this study, interpreting analysis results with caution is warranted, as they may not be applicable to other STEM intervention programs across the nation.

## Results

### Characteristics of H-LSAMP Participants

Descriptive results of the socio-demographic and pre-college characteristics for the 1,767 H-LSAMP scholars included in the sample are presented in Table 1. Approximately 36% of H-LSAMP participants in the sample were Black and 18.8% were Hispanic. The sample contained a higher proportion of male students (54.6%) compared to female (45.4%). Students who participated in the workforce while pursuing their undergraduate degree comprised 34% of the sample. The average total SAT score for students in the sample was nearly 681. There were approximately 500 missing values for standardized test scores, therefore these results should be interpreted with caution as not all students' SAT scores were included in the data available for this study.

**Table 1:** Descriptive statistics–H-LSAMP scholars.

**Variable**	**N %**
**Race**	
White	322 18.2
Black	627 35.5
Hispanic	332 18.8
Asian	343 19.4
Other/unknown	143 8.1
**Gender**	
Male	965 54.6
Female	802 45.4
**Workforce participation**	
No	1,166 66.0
Yes	601 34.0
**SAT total score**	μ = 681.4 σ = 574.7
**College acceptance status**	
Accept based on top 10%	508 28.8
Accept based on top 25%	238 13.5
Accept after meeting requirement	109 6.2
Accept based on other criteria	908 51

Approximately 42% of students in the sample were admitted into college based on merit status, including the top 10% and top 25% admission criteria. Students accepted based on other criteria comprised the majority of the sample (51%), while students who met the basic requirement for admission represented the smallest percentage of HLSAMP scholars (6.2%).

Table 2 demonstrates the proportion of students participating in the workforce while active in the H-LSAMP program across race/ethnic background. Black students represented the highest proportion of students simultaneously participating in the workforce and the H-LSAMP program (37.6%), while Hispanic students represented 17.6% of simultaneous workforce participation.

**Table 2:** Workforce participation across race/ethnic background.

**Workforce participation**	**White** ***N*** **%**	**Black** ***N*** **%**	**Hispanic** ***N*** **%**	**Asian** ***N*** **%**	**Other/unknown** ***N*** **%**
No	206 17.7	401 34.4	226 19.4	237 20.3	96 8.2
Yes	116 19.3	226 37.6	106 17.6	106 17.6	47 7.8
Total	322 18.2	627 35.5	332 18.8	343 19.4	143 8.1

Table 3 presents chi-square test results measuring the significance of differences across students who participated in the workforce while studying, and graduation. Results indicated a significant Pearson chi-square value (*p* < 0.005), demonstrating that a considerably lower proportion of students participating in the workforce during the H-LSAMP program graduated (59.2%), compared to students who did not work (98.1%). It is important to note that a proportion of students in the sample, specifically those graduating in 2015, may not have yet graduated given the six-year graduation timeframe.

**Table 3:** Chi-square test – differences in workforce participation and graduation among H-LSAMP participants.

**Workforce participation**	**Graduated** ***N*** **%**	**Not graduated** ***N*** **%**
No	1,144 98.1	22 1.9
Yes	356 59.2	245 40.8
Total	378 21.4	1,389 78.6

*Pearson Chi^2^ = 775.62. P < 0.005*.

### Workforce Participation Effects on Graduation

Table 4 presents results from the logistic regression model measuring the effect of workforce participation on graduation across H-LSAMP participants, along with corresponding coefficients and confidence intervals. Results indicated a statistically significant model with a pseudo R2 value of 0.467, indicating a considerable amount of variance in graduation rates among H-LSAMP participants (46.7%) could be explained by the independent variables included in the model. Generally, findings from the model showed that race, workforce participation and admissions criteria had significant associations with the graduation rates of H-LSAMP participants. Specifically, results showed that Black students had a significantly lower chance of graduating compared to their White peers (*p* < 0.05). Students accepted under other criteria had a significantly lower probability of graduating compared to those accepted under the 10% plan (*p* < 0.005). Finally, model results indicated a significant negative association between workforce participation while studying and graduation rates among H-LSAMP participants (*p* < 0.005).

**Table 4:** Logistic regression results–effect of workforce participation on graduation of H-LSAMP scholars.

**Variables**	**Coefficient**	**95% C.I**	
Race (white^1^)			
Black	−0.85^**^	−1.30	−0.41
Hispanic	−0.37	−0.88	0.14
Asian	0.53	0.005	1.05
Unknown/other	−0.049	−0.70	0.61
Gender (male^1^)			
Female	0.11	−0.21	0.43
Acceptance criteria (accepted under top 10%^1^)			
Accepted based on top 25%	−0.45	−0.96	0.069
Accepted, met criteria	−0.44	−1.17	0.28
Accepted, not met criteria	0.67	−2.09	3.43
Accepted based on other criteria	0.53^**^	−0.91	−0.16
Workforce participation (non-participant^1^)			
Workforce participant	−4.47		
Model summary	1,767		
N			
Nagelkerke R^2^	0.468		
−2 Log likelihood (df)	−488.19		

1 *refers to the reference group*.

** *refers to significance at the 0.005 level*.

## Discussion

Findings from this analysis corroborate previous research studies that underscore the effect of financial aid on college completion in STEM, particularly for under-represented students. In particular, results imply that workforce participation during the H-LSAMP program, despite the financial aid provided for taking part in this STEM success initiative, is negatively associated with the successful graduation of H-LSAMP scholars. In addition, our findings demonstrate that Black students participate in the workforce during their H-LSAMP participation to a greater extent than their peers and are significantly less likely to graduate compared to peers from other racial backgrounds. These results suggest that students from historically under-served backgrounds, specifically Black students who participate in the H-LSAMP program, face a greater need to find other sources of income to fund their education. As referenced in prior studies (e.g., Gardner and Broadus, 1990; Curs and Harper, 2012), minority students are often at a greater disadvantage to succeed in a STEM degree given the financial strain of affording their college education.

The financial concerns of this subpopulation of students, in addition to the cumulative disadvantage they already face due to inadequate schooling and barriers to access higher education, deter their chances of succeeding in an undergraduate STEM field of study (Blustein et al., 2013; Mitchell et al., 2019). Along those lines, results from this study support the notion that the financial aid offered through STEM intervention programs may not be sufficient to overcome the level of financial need these students face, leaving them less time to overcome the gaps in their academic preparation as they engage in the workforce to attend college. Given that one of the objectives of STEM intervention programs is to expand and diversify access of historically under-served students into STEM fields, these results offer evidence to revisit the level of financial aid provided to students and institutional policies that essentially run counter to efforts to broaden access into STEM. As prior scholars have highlighted, findings from the current study provide evidence for policy makers to strengthen need-based aid programs, rather than merit-based programs, that would be most beneficial to students with high financial need, as well as create funding formulas centralizing on capacity building for students with the least resources to pursue a degree in higher education (Mitchell et al., 2019). Particularly as it relates to decreases in higher education funding, along with tuition increases and the recent stagnation in earnings, these recommended shifts in policies would have a profound effect on reducing racial disparities in higher education attainment (Dynarski, 2003; Stater, 2009; Curs and Harper, 2012). In addition to the recommended changes to financial aid policy, both high schools and higher education institutions are encouraged to work with low-income minority students and their families to assist them in understanding the costs associated with pursuing a degree and the amount of expected financial aid they would receive upon acceptance into the HLSAMP program (Melguizo and Chung, 2012). Given the increased likelihood of low-SES students to undergo debt and pursue employment to cover their education costs, an increased understanding of the costs involved throughout their degree would be helpful in terms of managing expectations related to degree affordability (Grodsky and Jones, 2007). This is particularly helpful in an era where public institutions are struggling with decreased funding and a loss of endowments, which reduces the probability of stronger financial aid packages for low-income students (Melguizo and Chung, 2012). To the extent that the current role of higher education centralizes on creating a level playing field for students from disadvantaged backgrounds, our findings offer legitimacy for STEM intervention programs, and the institutions running such programs, to re-evaluate their admission criteria in ways that offer expanded opportunities for students most in need, both academically and financially. Re-evaluation criteria should include enhanced academic support for those students from disadvantaged backgrounds, particularly in introductory math and science courses, in order to strengthen their foundational academic skills that will enable them to successfully complete a degree in STEM. Furthermore, admission criteria should offer expanded access to this subpopulation of students, particularly through a shift in institutional admission criteria from merit-based to need-based aid. Such shifts will bolster the financial aid offered to disadvantaged students, and create a more balanced playing field where these students are offered equitable access to higher education as their peers from more affluent financial backgrounds.

### Directions for Future Research

In light of the evidence presented in this study that demonstrate the significant negative association between work-study and degree completion rates, an area for future research could build on this these findings and investigate differentiating effects of various occupations on degree completion. For instance, the degree to which working in a STEM field, vs. a non-STEM field, while studying effects graduation. In addition, examining the extent to which on-campus vs. off-campus employment affects degree completion rates could also provide valuable information to program leaders on whether providing paid on-campus job opportunities as an option could benefit the academic success of HLSAMP students. This level of detail could offer program administrators recommendations on guided career preparation and career resources that enable them to pursue an occupation that is relevant to their degree and could potentially promote higher graduation rates among URM students. Given the lack of research available in the area of workforce participation and its association with degree completion, such analyses would contribute to the discourse on the need for further financial support of students from disadvantaged/under-represented backgrounds (Alon and Tienda, 2007; Curs and Harper, 2012).

## Data Availability Statement

The raw data supporting the conclusions of this article will be made available by the authors, without undue reservation.

## Ethics Statement

The studies involving human participants were reviewed and approved by Institutional Review Board. The patients/participants provided their written informed consent to participate in this study.

## Author Contributions

DG conducted the analyses for this manuscript and prepared the first draft. DP and CH made significant intellectual contributions to the development and editing of the manuscript. DP was involved in the operation of the STEM Enrichment Program at the University of Houston during 2014 and 2015 and currently oversees the program. All authors approved the final version of the manuscript for publication.

## Funding

The research was funded by the National Science Foundation (NSF) Louis Stokes Alliance for Minority Participation. The NSF # for the award dated, 9/1/19–2/28/24, is HRD-911310(HRD# 000177721). The NSF # for the award dated, 9/1/14–2/28/21, is HRD-1407736(HRD# G107891). The NSF #for the award dated, 9/1/09–8/31/15, is HRD-0903948(HRD#G097982).

## Conflict of Interest

The authors declare that the research was conducted in the absence of any commercial or financial relationships that could be construed as a potential conflict of interest.

## Publisher's Note

All claims expressed in this article are solely those of the authors and do not necessarily represent those of their affiliated organizations, or those of the publisher, the editors and the reviewers. Any product that may be evaluated in this article, or claim that may be made by its manufacturer, is not guaranteed or endorsed by the publisher.

## References

[B1] Alon S. Tienda M. (2007). Diversity, opportunity, and the shifting meritocracy in higher education. Am. Sociol. Rev. 72, 487–511. 10.1177/000312240707200401

[B2] Aschbacher P. R. Li E. Roth E. J. (2010). Is science me? High school students' identities, participation and aspirations in science, engineering, and medicine. J. Res. Sci. Teach. 47, 564–582. 10.1002/tea.20353

[B3] Baber L. D. (2015). Considering the interest-convergence dilemma in STEM education. Rev. Higher Educ. 38, 251–270. 10.1353/rhe.2015.0004

[B4] Blustein D. L. Barnett M. Mark S. Depot M. Lovering M. Lee Y. DeBay D. (2013). Examining urban students' constructions of a STEM/career development intervention over time. J. Career Develop. 40, 40–67. 10.1177/0894845312441680

[B5] Bonsangue M. V. Drew D. E. (2021). The Houston-Louis Stokes Alliance for Minority Participation Evaluation Report. Houston, TX: United states. The University of Houston.

[B6] Bowen W. G. Chingos M. M. McPherson M. S. (2009). Crossing the Finish Line. Princeton University Press. 10.1515/9781400831463

[B7] Cabrera A. F. Nora A. Castaneda M. B. (1993). College persistence: Structural equations modeling test of an integrated model of student retention. J. High. Educ. 64, 123–139. 10.1080/00221546.1993.11778419

[B8] Carnevale A. P. Smith N. Melton M. (2011). STEM: Science Technology Engineering Mathematics. Georgetown University Center on Education and the Workforce. Available online at: https://files.eric.ed.gov/fulltext/ED525297.pdf

[B9] Carpi A. Ronan D. M. Falconer H. M. Lents N. H. (2017). Cultivating minority scientists: Undergraduate research increases self-efficacy and career ambitions for underrepresented students in STEM. J. Res. Sci. Teach. 54, 169–194. 10.1002/tea.21341

[B10] Chang M. J. Sharkness J. Hurtado S. Newman C. B. (2014). What matters in college for retaining aspiring scientists and engineers from underrepresented racial groups. J. Res. Sci. Teach. 51, 555–580. 10.1002/tea.21146

[B11] Chapa J. De La Rosa B. (2006). The problematic pipeline: Demographic trends and Latino participation in graduate science, technology, engineering, and mathematics programs. J. Hispanic Higher Educ. 5, 203–221. 10.1177/1538192706288808

[B12] Cheeseman Day J. Martinez A. (2021). STEM Majors Earned More Than Other STEM Workers. Available online at: https://www.census.gov/library/stories/2021/06/does-majoring-in-stem-lead-to-stem-job-after-graduation.html

[B13] Chen X. Weko T. (2009). Stats in Brief: Students Who Study Science, Technology, Engineering, and Mathematics (STEM) in Postsecondary Education. Washington, DC: United States. Vol. 161. Available online at: https://files.eric.ed.gov/fulltext/ED506035.pdf

[B14] Crisp G. Nora A. Taggart A. (2009). Student characteristics, pre-college, college, and environmental factors as predictors of majoring in and earning a STEM degree: an analysis of students attending a Hispanic serving institution. Am. Educ. Res. J. 46, 924–942. 10.3102/0002831209349460

[B15] Curs B. R. Harper C. E. (2012). Financial aid and first-year collegiate GPA: A regression discontinuity approach. Rev. High. Educ. 35, 627–649. 10.1353/rhe.2012.0040

[B16] Dika S. L. D'Amico M. M. (2016). Early experiences and integration in the persistence of first-generation college students in STEM and non-STEM majors. J Res. Sci. Teach. 53, 368–383. 10.1002/tea.21301

[B17] Doyle W. R. (2006). Community college transfers and college graduation: whose choices matter most? Change: Mag. High. Learn. 38, 56–58. 10.3200/CHNG.38.3.56-58

[B18] Duncan H. Dick T. (2000). Collaborative workshops and student academic performance in introductory college mathematics courses: A study of a Treisman model math excel program. School Sci. Mathem. 100, 365–373. 10.1111/j.1949-8594.2000.tb18178.x

[B19] Dynarski S. M. (2003). Does aid matter? Measuring the effect of student aid on college attendance and completion. Am. Econ. Rev. 93, 279–288. 10.1257/000282803321455287

[B20] Edge L. (2020). Science has a racism problem. Cell. 181, 1443–1444. 10.1016/j.cell.2020.06.009 32521231

[B21] Engberg M. Wolniak G. (2013). College student pathways to the STEM disciplines. Teach. College Rec. 115, 1–27. 10.1177/016146811311500102

[B22] Espinosa L. (2011). Pipelines and pathways: Women of color in undergraduate STEM majors and the college experiences that contribute to persistence. Harv. Educ. Rev. 81, 209–241. 10.17763/haer.81.2.92315ww157656k3u

[B23] Estrada M. (2014). Ingredients for improving the culture of STEM degree attainment with co-curricular supports for underrepresented minority students. National Academies of Sciences White Paper, 28. Available online at: https://sites.nationalacademies.org/cs/groups/dbassesite/documents/webpage/dbasse_088832.pdf

[B24] Fenske R. H. Porter J. D. Dubrock C. P. (2000). Tracking financial aid and persistence of women, minority, and needy students in science, engineering, and mathematics. Res. High. Educ. 41, 67–94. 10.1023/A:1007042413040

[B25] Gardner P. D. Broadus A. (1990). Pursuing an Engineering Degree: An examination of issues pertaining to persistence in Engineering. East Lansing: Michigan State University Collegiate Employment Research Institute.

[B26] Ghazzawi D. Pattison D. Horn C. (2021b). Persistence of underrepresented minorities in STEM fields: are summer bridge programs sufficient? Front. Educ. 6, 224. 10.3389/feduc.2021.630529

[B27] Ghazzawi D. Pattison D. L. Horn C. Hardy J. Brown B. (2021a). Impact of an intensive multi-disciplinary STEM enrichment program on underrepresented minority student success. J. Appl. Res. High. Educ. 10.1108/JARHE-12-2020-0452

[B28] Ghee M. Keels M. Collins D. Neal-Spence C. Baker E. (2016). Fine-tuning summer research programs to promote underrepresented students' persistence in the STEM pathway. CBE—Life Sci. Educ. 15, ar28. 10.1187/cbe.16-01-0046 27496359 PMC5008875

[B29] Grodsky E. Jones M. T. (2007). Real and imagined barriers to college entry: perceptions of cost. Soc. Sci. Res. 36, 745–766. 10.1016/j.ssresearch.2006.05.001

[B30] Institute For Broadening Participation (2014). Designing for Success. Available online at: http://www.ibparticipation.org/pdf/Designing_for_Success.pdf

[B31] Jackson K. M. Winfield L. L. (2014). Realigning the crooked room: spelman claims a space for African American women in STEM. Peer Rev. 16, 9. 25558184 PMC4280840

[B32] Lane T. B. (2016). Beyond academic and social integration: Understanding the impact of a STEM enrichment program on the retention and degree attainment of underrepresented students. CBE—Life Sci. Educ. 15, ar39. 10.1187/cbe.16-01-0070 27543638 PMC5008886

[B33] Lee D. M. Harmon K. (2013). “The Meyerhoff Scholars Program: Changing Minds”, in Transforming a Campus. Metropolitan Universities, vol. 24, p. 55–70. Available online at: https://journals.iupui.edu/index.php/muj/article/view/20547

[B34] Lisberg A. Woods B. (2018). Mentorship, mindset and learning strategies: An integrative approach to increasing underrepresented minority student retention in a STEM undergraduate program. J. STEM Educ. 19, 14–20. Available online at: https://www.learntechlib.org/p/184625/

[B35] Ma J. Matea P. (2021). Trends in College Pricing and Student Aid 2021. New York, NY: College Board. Available online at: https://research.collegeboard.org/pdf/trends-college-pricing-student-aid-2021.pdf

[B36] May G. S. Chubin D. E. (2003). A retrospective on undergraduate engineering success for underrepresented minority students. J. Eng. Educ. 92, 27–39. 10.1002/j.2168-9830.2003.tb00735.x28040097

[B37] McGee E. O. (2020). Interrogating structural racism in STEM higher education. Educ. Res. 49, 633–644. 10.3102/0013189X20972718

[B38] Melguizo T. Chung A. (2012). College aid policy and competition for diversity. Rev. High. Educ. 35, 403–430. 10.1353/rhe.2012.0021

[B39] Mitchell M. Leachman M. Saenz M. (2019). State Higher Education Funding Cuts Have Pushed Costs to Students, Worsened Inequality. Washington, DC: Center on Budget and Policy Priorities. Available online at: https://tacc.org/sites/default/files/documents/2019-11/state_he_funding_cuts.pdf

[B40] National Assessment of Educational Progress (2011). The nation's report card: The official site for results from the National Assessment of Educational Progress (NAEP). Grade 12 National science results 2009. Available online at: http://nationsreportcard.gov/science_2009/g12_nat.asp?subtab_idTab_1andtab_idtab2#tabsContainer

[B41] National Center for Science Engineering Statistics (2018). “Science and Engineering Degrees, by Race and Ethnicity of Recipients.” Available online at: https://www.nsf.gov/statistics/degreerecipients/

[B42] O'Hara R. M. (2020). STEM (ing) the tide: a critical race theory analysis in STEM education1. J. Constr. Psychol. 1:1–13. 10.1080/10720537.2020.1842825

[B43] Riegle-Crumb C. King B. (2010). Questioning a white male advantage in STEM: examining disparities in college major by gender and race/ethnicity. Educ. Res. 39, 656–664. 10.3102/0013189X10391657

[B44] Rincón B. E. George-Jackson C. E. (2016). STEM intervention programs: funding practices and challenges. Studies High. Educ. 41, 429–444. 10.1080/03075079.2014.927845

[B45] Rincón B. E. Lane T. B. (2017). Latin@ s in science, technology, engineering, and mathematics (STEM) at the intersections. Equity Excell. Educ. 50, 182–195. 10.1080/10665684.2017.1301838

[B46] Smyth F. L. McArdle J. J. (2004). Ethnic and gender differences in science graduation at selective colleges with implications for admission policy and college choice. Res. High. Educ. 45, 353–381. 10.1023/B:RIHE.0000027391.05986.79

[B47] Stater M. (2009). The impact of financial aid on college GPA at three flagship public institutions. Am. Educ. Res. J. 46, 782–815. 10.3102/0002831208329903

[B48] Thomas S. L. (2000). Deferred costs and economic returns to college major, quality, and performance. Res. High. Educ. 41, 281–313. 10.1023/A:1007003510102

[B49] Treisman U. (1992). Studying students studying calculus: a look at the lives of minority mathematics students in college. Coll. Mathem. J. 5, 362–72. 10.1080/07468342.1992.11973486

[B50] Trent W. T. Lee H. S. Owens-Nicholson D. (2006). Perceptions of financial aid among students of color: examining the role (s) of self-concept, locus of control, and expectations. Am. Behav. Sci. 49, 1739–1759. 10.1177/0002764206289146

[B51] Walker K. George-Jackson C. Rincon B. Williams M. Baber L. Trent W. (2010). “STEM Intervention Programs: The Shift from Opportunity to Merit” in Association of the Study of Higher Education Conference Paper. Indianapolis, Indiana. Available online at: https://citeseerx.ist.psu.edu/viewdoc/download?doi=10.1.1.1063.9528andrep=rep1andtype=pdf

[B52] Xie Y. Fang M. Shauman K. (2015). STEM education. Ann. Rev. Sociol. 41, 331–357. 10.1146/annurev-soc-071312-145659 26778893 PMC4712712

[B53] Xie Y. Killewald A. A. (2012). Is American Science in Decline? Harvard University Press. 10.4159/harvard.9780674065048

